# An evaluation of a novel method for the MRI-based assessment of Caton-Deschamps index in the Knee

**DOI:** 10.1007/s00402-024-05403-5

**Published:** 2024-06-20

**Authors:** Yannick Palmowski, Tobias Jung, Sarah Hellwig, Stephan Oehme, Stephen Fahy, Benjamin Bartek

**Affiliations:** Center for Musculoskeletal Surgery, Charité – Universitätsmedizin Berlin, corporate member of Freie Universität Berlin, Humboldt-Universität zu Berlin, Berlin Institute of Health, Chariteplatz 1, 10117 Berlin, Germany

**Keywords:** Patellar instability, MRI methods, Caton-Deschamps index, Patella alta

## Abstract

**Introduction:**

The radiographical assessment of patella height has historically been performed using X-Ray. The aim of this study was to evaluate a new method for the assessment of patella height using MRI and to assess the correlation with the X-Ray based assessment.

**Materials and methods:**

159 patients who had both lateral radiographs and MRI images were included. Parameters measured included traditional radiographical CDI, MRI-based CDI, and TT-TG distance. On the basis of the TT-TG, the patients were divided into 2 groups. Two different methods were used to assess CDI using MRI: using a single slice image, and an alternative technique using two different cross-sectional images. The correlation of the two measurement methods was assessed using Pearson’s correlation coefficient. The intraclass correlation coefficient (ICC) was determined from the measurements of the two investigators.

**Results:**

The average TT-TG distance was 11.6 mm (± 4.6). In patients with a TT-TG < 15 mm, both measurement methods showed comparable correlation with measurements on X-Ray. In patients with a TT-TG of > 15 the the new cross-sectional imaging method showed higher correlation with traditional X-Ray assessment compared to CDI assessment using the traditional single slice method (*r* = 0.594, *p* < 0.001 vs. *r* = 0.302, *p* = 0.055).

**Conclusions:**

The assessment of CDI on MRI using a cross-sectional imaging method has a better correlation with traditional X-Ray assessment of CDI than single-slice assessment. This is particularly true in patients with elevated TT-TG and as such should be preferentially used in the assessment of Patellar height in this cohort.

## Introduction

Patellar dislocation is a common condition, with an annual incidence of 5.8 per 100,000 people. Patellar dislocation can lead to recurrent instability which has a large associated morbidy [1, 2].

Patella alta is one of the main risk factors for lateral patellar dislocation (LPD) [1, 3, 4]. Several methods can be used to determine and quantify patella height. The most commonly used measurements include; the Insall-Salvatti index (IS), the Blackburne-Peel index (BP) and Caton-Deschamps index (CD) [5–7]. These measurements are made using lateral Knee X-Rays [8–11].

Magnetic resonance imaging (MRI) is vital following LPD, it allows for assessment of cartilage morphology and ligamentous disruption. Additionally, MRI allows for a thorough assessment of bony morphology including; trochlear dysplasia, increased tibial tuberosity and trochlear groove distance (TT-TG), and the presence of patella alta, all of which are predictors of persistent patellar instability. Although MRI assessment is widely accepted as the gold standard for diagnostic imaging following LPD, some studies have suggested that the assessment of patellar height in MRI correlates poorly with the traditional measurements mentioned above [4, 12, 13].

The MRI based assessment of patellar height is usually performed using a single-slice sagittal view. We hypothesized that the single-slice assessment of patellar height would be poorly correlated with traditional X-Ray based CD assessment in patients with an increased TT-TG owing to the lateralization of the patella which makes it difficult to view the tibial tuberosity and the patella on one sagittal image.

The purpose of our study is; (1) to compare the accuracy of CD measurements on MR-images compared to lateral X-Ray images in patients with normal and increased TT-TG distance (2) to evaluate the accuracy of a new two-slice method for the assessment of CD on MRI and compare it against the standard single-slice approach.

Our primary hypothesis is that single-slice assessment of CD on MRI is poorly correlating with traditional X-Ray-based CD in patients with increased TT-TG as compared to patients with normal TT-TG values. Our secondary hypothesis is that a new two slice assessment method has a higher correlation with X-Ray-derived CD than the single-slice technique.

## Materials and methods

### Patients

159 consecutive patients with a history of either medial patellofemoral ligament or anterior cruciate ligament reconstruction in our institution between the dates 07/2019-06/2021 were included in the study. Only patients with both lateral radiographic images and MRI scans of the affected knee were included. Patients with insufficient or incomplete images were excluded from this study. No further exclusion criteria were applied. Patients were divided into groups with TT-TG < 15 mm and TT-TG ≥ 15 mm. In keeping with international best practice, MRI’s were performed with the knee in almost full extension, while lateral knee radiographs were performed in a lying position, with an approximate 135° angle between femur and tibia.

### Data collection

Two readers independently assessed all X-Ray and MR-images for the relevant radiographic parameters. Data for this research were accessed during the period from 03/2022 to 05/2022. Image analysis was carried out using Phönix PACS Version 7.0 (Phönix-PACS GmbH, Freiburg, Germany).

**Fig. 1:**
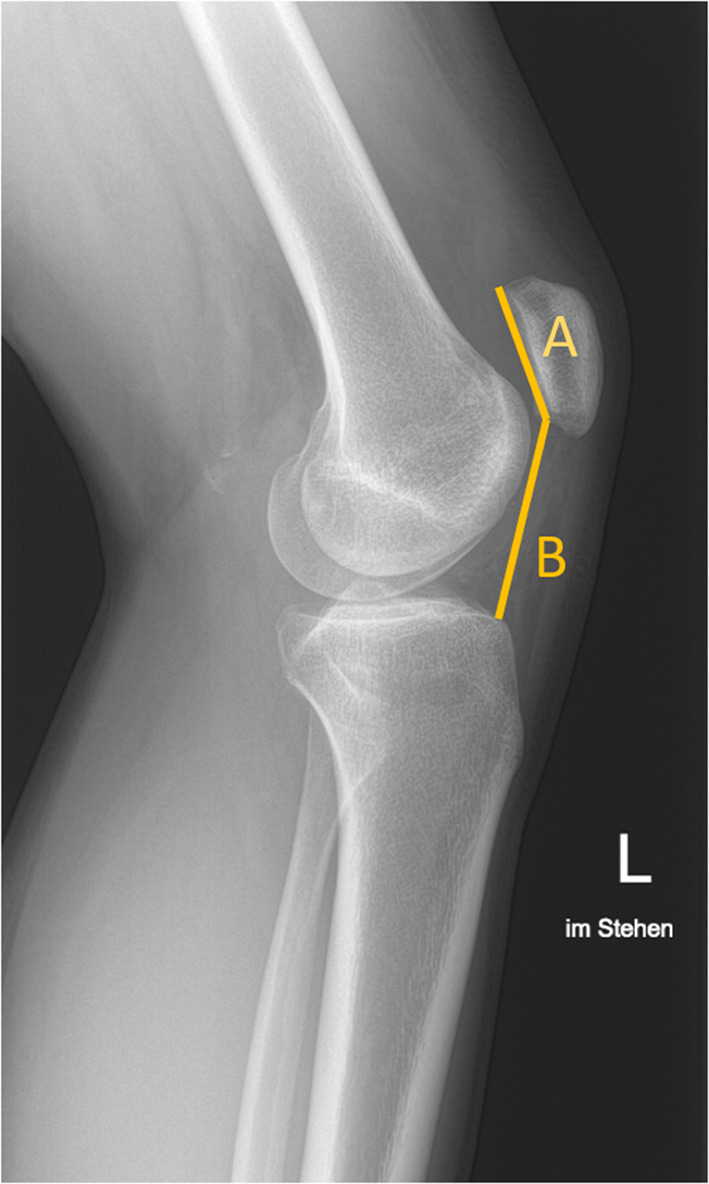
Conventional X-Ray lateral view. Cato–Deschamps (CD) index measurement. A patellar length, B distance from the patella to the tibia. CD: B/A

The traditional CD assessment of patellar height was performed on lateral X-rays (Fig. 1) [10, 14]. The CD is obtained by measuring two different parameters; (1) the length of the articular surface of the patella and (2) the distance between the inferior aspect of articular surface of the patella and the anterior angle of the tibial plateau.

**Fig. 2:**
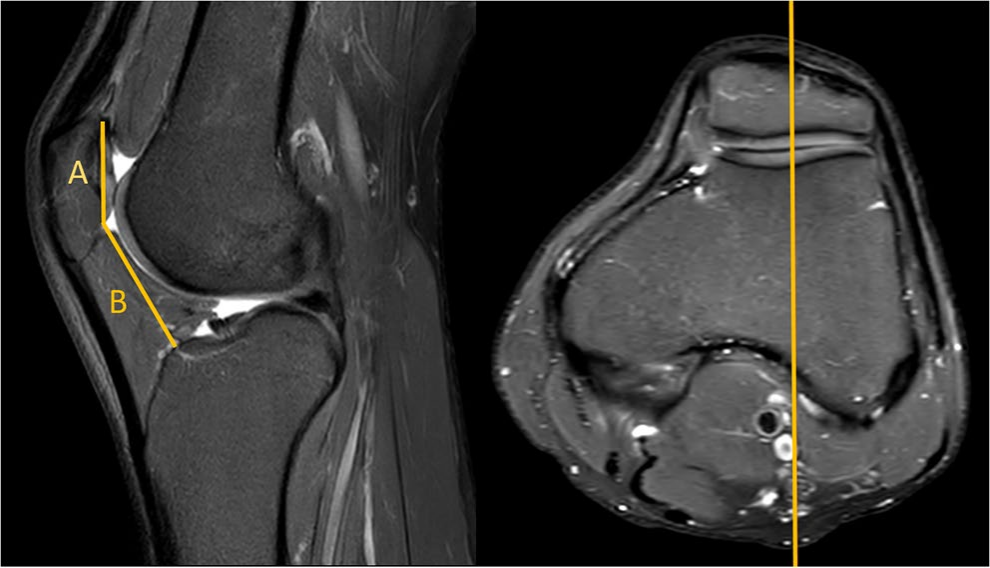
MRI true sagittal view. Caton–Deschamps (CD) index measurement with one slice. B distance from the patella to the tibia and A patellar cartilage length. CD: B/A

**Fig. 3:**
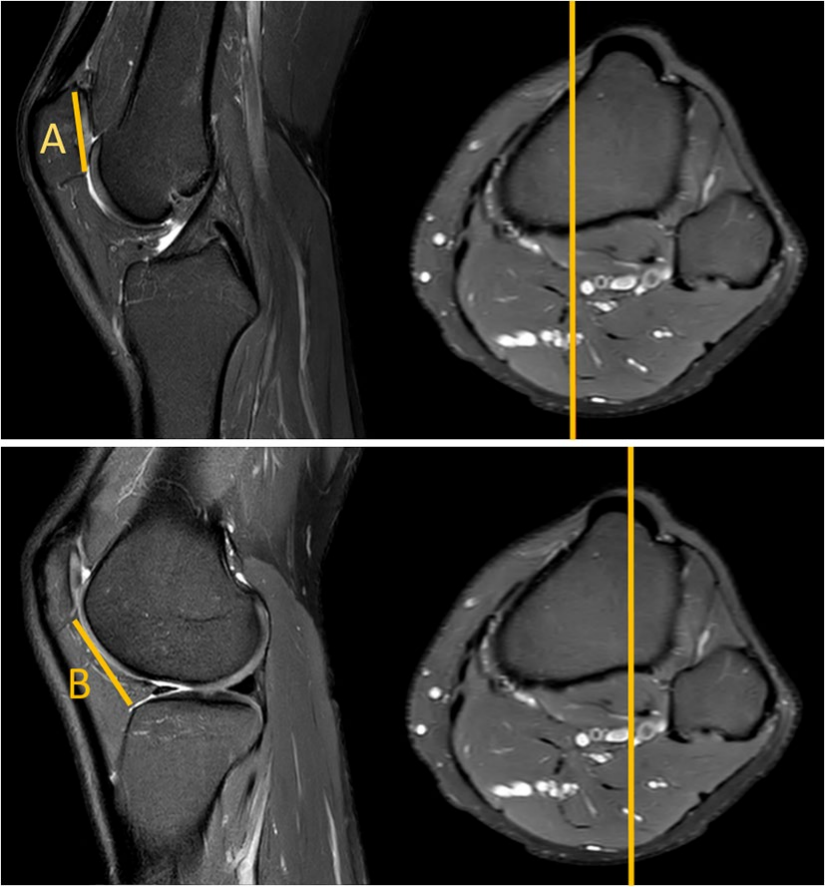
MRI true sagittal view. Caton–Deschamps (CD) index measurement in two slices. A patellar cartilage length (measured in slice with the maximum length of the articular surface of the patella) B distance from Line A to the tibia (measured in slice going through the centre of the tibial tuberosity). CD: B/A

The MRI-based assessment of CD was performed using both the standard single-slice method, and the new two-slice method. The single slice assessment of CD was performed on a single sagital MR-images, utilizing the plane with the maximum length of the articular surface of the patella (Fig. 2). The two-slice method firstly measured the length of the articular surface of the patella at its maximum length (similar to Method 1), and then assessed the distance to the anterior angle of the tibial plateau in the sagittal plane going through the center of the tibial tuberosity (Fig. 3).

Additionally, the TT-TG distance was measured in all patients using the traditional assessment method.

### Statistical analysis

The mean values for the measurements from both reviewers were calculated and used for statistical analysis. Mean values with standard deviation (SD) were calculated for descriptive statistics. As the data was normally distributed, Pearson correlation coefficient was used to evaluate the correlation between X-Ray and MRI measurements. The inter-reader reliability for each method was assessed using the intra-class correlation coefficient (ICC).

### Results

The mean age of the patient was 32.2 (± 10.4) years (Table 1). The mean TT-TG was 11.6 mm (± 4.6), with a mean CD on X-Ray of 1.05 (± 0.18). Mean CD values on MRI were slightly higher with 1.11 (± 0.18) for the single-slice measurement and 1.11 (± 0.16) for the two-slice measurement. The correlation between MRI based and X-ray based assessment was 0.451 (*p* < 0.001) for the single-slice MRI assessment and 0.558 (*p* < 0.001) for the two-slice MRI assessment. ICC was 0.678 (*p* < 0.001) for MRI measurements on a single plane, 0.823 (*p* < 0.001) for the two-slice assessment method and 0.866 (*p* < 0.001) for X-Ray measurements.

**Table 1:** Descriptive statistics

	Minimum	Maximum	Mean	SD
Age	15.00	71.00	32.1698	10.35460
TT-TG	2.46	23.92	11.5972	4.60921
CD (MRI 1 plane)	0.70	1.74	1.1062	0.17707
CD (MRI 2 planes)	0.75	1.61	1.1112	0.16176
CD (X-Ray)	0.47	1.74	1.0477	0.17858

TT-TG: distance between tibial tuberosity and trochlear groove, CD: Caton-Deschamps Index; SD: standard deviation

### Patients with TT-TG < 15 mm

118 patients had TT-TG values of < 15 mm. Descriptive statistics for these patients are presented in Table 2. For these patients, the correlation with the measurement on X-ray images was 0.497 (*p* < 0.001) for the MRI measurement on a single slice and 0.521 (*p* < 0.001) for the MRI measurement using the two-slice method.

ICC was 0.62 (*p* < 0.001) for MRI measurements on a single plane, 0.772 (*p* < 0.001) using the two-slice method, and 0.874 (*p* < 0.001) for X-Ray measurements.

**Table 2:** Descriptive statistics for patients with TT-TG < 15 mm

	Minimum	Maximum	Mean	SD
Age	15.00	71.00	33.0085	10.80321
TT-TG	2.46	14.77	9.4015	2.77705
CD (MRI 1 plane)	0.70	1.66	1.0817	0.16239
CD (MRI 2 planes)	0.75	1.44	1.0904	0.15078
CD (X-Ray)	0.47	1.43	1.0321	0.17010

TT-TG: distance between tibial tuberosity and trochlear groove, CD: Caton-Deschamps Index; SD: standard deviation

### Patients with TT-TG ≥ 15 mm

41 patients had TT-TG values of < 15 mm. Descriptive statistics for these patients are presented in Table 3. For these patients, the correlation with the measurement on X-Ray images was 0.302 (*p* < 0.055) for the single-slice MRI measurement and 0.594 (*p* < 0.001) for the two-slice method.

ICC was 0.767 (*p* < 0.001) for MRI measurements on a single plane, 0.901 (*p* < 0.001) for MRI measurements using the two-slice method and 0.841 (*p* < 0.001) for X-Ray measurements.

**Table 3:** Descriptive statistics for patients with TT-TG ≥ 15 mm

	Minimum	Maximum	Mean	SD
Age	16.00	55.00	29.7561	8.60750
TT-TG	15.04	23.92	17.9165	2.49112
CD (MRI 1 plane)	0.86	1.74	1.1767	0.19961
CD (MRI 2 planes)	0.87	1.61	1.1710	0.17863
CD (X-Ray)	0.72	1.74	1.0927	0.19632

TT-TG: distance between tibial tuberosity and trochlear groove, CD: Caton-Deschamps Index; SD: standard deviation

## Discussion

This study found that MRI based assessment of CD generally yields higher values than the traditional X-Ray based assessment, a finding in keeping with those seen in previous studies [13, 15, 16]. This is likely attributable to variations in patient positioning during both MRI and X-Ray. MRI is usually performed with the knee in almost full extension, whereby the clinical standard for lateral X-Rays is an angle of 135° between femur and tibia. This inevitably influences the position of the patella and may therefore also have an effect on the assessment of patellar height. Despite this, a significant correlation was found between X-Ray and MRI assessment methods [17].

MRI based assessment of CD has traditionally been performed using a single-slice assessment method. We found that in patients with an increased TT-TG single-slice assessment of CD had a significantly lower correlation to traditional X-Ray based assessment (*r* = 0.302, *p* < 0.055) than in patients with a normal TT-TG (*r* = 0.497, *p* < 0.001), which confirmed our primary hypothesis. This result is extremely important for the interpretation of CD values based on MRI images. The CD is mainly used when a patella alta is suspected, which is a risk factor for LPD [1–3, 5, 14]. An increased TT-TG is another common risk factor for LPD and both conditions are often seen simultaneously in this patient cohort [2, 3, 18]. Therefore, CD measurements using only the single-slice technique in MRI could result in both an in-precise and misleading assessment of patellar height, which may negatively influence treatment decisions. Based on our research, we feel that this method should not be used in clinical practice.

A potential explanation for the influence of the TT-TG on the correlation between MRI and X-Ray based assessment of CD lies in the dynamic nature of MR-imaging. In X-Ray images, both the full length of the patella’s articular surface and the anterior angle of the tibial plateau, are reliably seen on the same image. In MRI scans however, these two landmarks will only appear on the same sagittal plane if the longest portion of the patellar articular surface is centered directly above the anterior angle of the tibial plateau. As the patella is usually centered in the trochlear groove and the anterior angle of the tibial plateau is located quite centrally over the tibial tuberosity, a low TT-TG increases the probability of both landmarks appearing in the same sagittal MRI plane. A high TT-TG on the other hand increases the risk of a lateralization of the patella, which will lead to the patella articular surface appearing in another sagittal plane than the anterior angle of the tibial plateau.

In order to account for the limitations of the single-slice MRI method, we developed an alternative two-slice method for the measurement of CD on MR-images. We aimed to reliably and reproducibly measure the CD similar to the way it would project in an X-Ray image. This method showed a much higher correlation with X-Ray measurements than the single slice method (*r* = 0.594, *p* < 0.001 vs. *r* = 0.302, *p* < 0.055). At the same time, this new method also showed excellent inter-reader reliability comparable to that of X-Ray measurements (ICC = 0.901, *p* < 0.001 and 0.841, *p* < 0.001, respectively). Therefore, we conclude that this method should preferably be used when evaluating patella height in MR-images.

The strengths of our study include a large patient cohort. In order to ensure a high clinical applicability, only patients undergoing knee surgery were included. A large percentage of our cohort had an increased TT-TG value, which was the group of interest for our research. In addition, we chose a robust study design, with two examiners independently assessing all images in order to reduce the risk of systematic bias.

However, our study has certain limitations. This is a retrospective single-center study. Furthermore, we only included the CD index as one important parameter of patellar height. Future studies will be needed to confirm the applicability of our results to other commonly used parameters such as Insall-Salvati or Blackburne-Peel ratio.

## Conclusion

MRI-based assessment of CD using the single-slice technique is not reliable in patients with increased TT-TG. Therefore, we advocate for a new method, proposed by this study to measure CD on MRI images. The assessment of CD using cross-sectional MRI imaging shows a strong correlation with traditional X-ray measurements and can, in certain cases serve as a reliable replacement for X-ray imaging. However, it is important to recognize the enduring value, broad applicability, and ease of interpretation of X-ray imaging, and as such it is unlikely that an MRI-based assessment of patellar height will replace traditional X-ray based assessment for the time being. Nevertheless, the two-slice method proposed herein demonstrates excellent inter-reader reliability across all patient cohorts, regardless of the TT-TG distance, and represents a valuable tool in the clinician’s diagnostic arsenal.

## Data Availability

The authors confirm that the data supporting the findings of this study are available within the article and its supplementary material. Raw data that support the findings of this study are available from the corresponding author, upon reasonable request.

## Abbreviations

BP: Blackburne-Peel index
CD: Caton-Deschamps index
ICC: intraclass correlation coefficient
IS: Insall-Salvatti index
LPD: lateral patellar dislocation
MRI: Magnetic resonance imaging
SD: standard deviation
TT-TG: tibial tuberosity and trochlear groove distance

## Declarations

### Critical relevance statement

Our article presents an innovative and reproducible MRI method for evaluating the Caton-Deschamps index in knee assessments. This breakthrough contributes significantly to the fields of orthopedics and radiology, offering practical applications for daily clinical practice.
